# On Stramonium

**Published:** 1811-05

**Authors:** 


### Medical and Physical Journal.

VOL. XXV.] MAY, 1811. [NO. 147.

Printed for R. PHILLIPS, by E. Hemsted, Great New Street, Fetter Lane, London.

### *For the Medical and Physical Journal.*

### *On Stramonium.*

WHEN the multitude are running after a new remedy, or an old one newly revived, experienced men are apt to say, "let them alone, they will soon be undeceived." Admitting the justice of the observation, I should not have written the following account of Stramonium; but having been induced, by the very general talk about its properties, to direct my attention to this subject, I collected some particulars respecting it, which I thought might not be uninteresting to the readers of this Journal. Before, however, I state the results of my inquiry, it may not be altogether useless to glance at the mode in which this remedy has recently been introduced to public notice. The first account of it which I have met with, is in the Monthly Magazine for June 1810, by a writer who signs himself VERAX. He describes himself to be what is termed a *bon-vivant*, and would appear to be possessed of Dr. Trotter's *nervous temperament;* he gives a pleasant account of his way of life, and details the symptoms of his asthmatic complaint in an easy popular style, a stile which the writers of *familiar* treatises on medicine might envy. Being checked in his luxurious indulgence, by spasmodic asthma, he found it necessary to consult "the most eminent physicians of the metropolis;" none of whom, however, afforded him "any thing more than a transient and tantalizing relief." At this juncture, he acquaints us—"An amiable friend and most respectable surgeon at Hackney, first persuaded me to smoke the divine Stramonium, to which I owe altogether my present freedom from pain, and renewed capacity of enjoyment. It is the root only, and lower part of the stem of this plant, which seem to possess its anti-asthmatic virtue: these should be cut into small pieces, and put into a common tobacco-pipe, and the smoke must be swal-

 lowed, lowed, together with the saliva produced by the smoke; after which the sufferer will, in a few minutes, be relieved from all the convulsive heavings, and probably drop into a comfortable sleep, from which he will awake refreshed; and, in general, perfectly recovered: at least, this is the invariable effect produced upon myself."

It is to be regretted that the name of the medical gentleman who recommended this *divine* remedy, is withheld from the public. Though the practice is known in the East Indies, I have in vain searched for authorities for smoking Stramonium in this country; at present, the honour of introducing it rests between Verax, and the author of a pamphlet which appeared precisely at the time, when the curiosity of asthmatics was sufficiently awakened by the accounts that had been inserted in the Monthly Magazine: they were thirsty, and a good Samaritan pointed to the well. The title of this pamphlet is, "A familiar Treatise on Asthma, Difficulty of Breathing, Wheezing, Winter Cough, and Consumption of the Lungs; containing, with other Information, explicit Directions for the Use of the Stramonium Herb, by Fisher, Surgeon, formerly an asthmatic Invalid." Manna could hardly have proved more grateful to the hungry Israelites, than this Treatise to the wheezing Asthmatics. The author's motive also was charitable; I shall quote it as being worthy of imitation. "The sole object the author had in this publication, was to communicate that information to those who suffered by affections of the lungs, which at one period of his life he required himself. The work having gone through two heavy editions in the short space of a month, is a proof how much such patients stood in need of advice; and the disappointment they, as well as himself, had experienced in their applications to a certain class of *regular* practitioners, whose end is attained, if they succeed in amusing the mind of their patients to enrich themselves."—(Pref. to 3d edit.)

But this gentleman's philanthropy is even surpassed by his modesty; he will not permit a patient, overflowing with gratitude, personally to express his thanks:—"The author understanding that several applications have been made to the publisher and printer of the work for his address, begs to observe, that he has omitted it in consequence of having no interested view in bringing his name before the public."—(Pref. iv.) His cover, indeed, is so impenetrable, that neither gentlemen nor poachers have yet discovered his form. But,

"A quoi sert cette mine modeste,
Et ce sage dehors, que dement tout le reste?"

Instead

Instead of containing a history of Stramonium, and a description of its effects, that might enable a practitioner to determine in what cases it might prove beneficial, this pamphlet gives a brief and imperfect delineation of some of the more prominent features of the complaints mentioned in the title, mingled with coarse abuse of *regular* practitioners, and at length informs us, "that the prepared Stramonium, and oxymel of it, may be obtained of," &c. &c.

"O curas hominum! O quantum est in rebus inane!

From what I have hinted, I think the design of this familiar treatise will be sufficiently obvious; and as it is not at present my intention to develop Mr. Fisher from his covering,

"And hew the block off, and get out the man,"

I shall proceed to the botanical description of the

*Datura Stramonium; common Thorn-Apple.*

Synonyma. Stramonium. *Pharm. Edin. Hall. helv. n.* 586. Solanum Fœtidum, pomo spinoso oblongo. *Bauh. Pin.* p. 168. Stramonium majus album. *Park. Parad.* p. 360. Stramonium Fœtidum. *Scop. carn. n.* 252. Stramonium Spinosum. *Gerard. Emac.* p. 348. *Raii Hist.* p. 748. Stramonium maniacum. *Col. phyt.* 47. Stramonium foliis angulosis, fructu erecto, muricato calyce pentagonia. *Hall Stirp. Helv. n.* 586. Datura Stramonium. *Lin. Spec.* 255. *Reich i.* 497. *Withering. Bot. Arrang.* p. 230. *Flor. Danic.* p. 436. *Stoerck. Libell de Stram. Curt. Flor. Lond. Gron. virg.* 33. *Pollich pal. n.* 224. *Krock. Siles. n.* 339. *Fl. Dan. t.* 436. *Blackw. t.* 313. *Sabb. hort. s. t.* 92. *Plenck. ic. t.* 96.

Class 5.1. Pentandria Monogynia. *Lin. Gen.* Plant. Nat. order of *Luridæ.*—Solaneæ Juss.

Generic Character.

Calyx. *Perianth,* one-leafed, oblong, tubular, bellied, five-cornered, five-toothed, horizontally deciduous near the base, the remaining circular part permanent.

Corolla. One-petalled, funnel-form. *Tube* greenish, cylindric, border erect expanding, five-cornered, five-plaited, almost entire, with five acuminate teeth.

Stamina. *Filaments* five, subulate, length of the calyx.

*Anthers* oblong, compressed obtuse, of a brownish yellow colour.

Pistillum. *Germ* ovate; *Style* white, filiform, straight; *Stigma* thickish obtuse, two-plaited.

Pericarp. *Capsule* somewhat ovate, two-celled, four-valved, seated on the base of the calyx. *Receptacles* convex, large, dotted, affixed to the dissepiment.

Seeds numerous, kidney-form.

The smoothness as well as the prickly roughness of the capsule in this genus varies.

### Essential Character.

*Cor.* funnel-form, plaited. *Cal.* tubular, angular, deciduous. *Caps.* four-valved.

Of this genus eight species are enumerated in Martyn's edition of Miller's dictionary.* The Datura Stramonium is

---

* Spec. 2. Datura ferox, *rough Thorn-Apple.* Syn. Stramonium ferox. S. longioribus aculeis. This is annual, and a native of China; was cultivated by Miller in 1731. Hort. Kew.

Spec. 3. Datura tatula, *blue Thorn-Apple.* Syn. Stramonium majus purpureum. Stem purplish with white dots; corolla pale blue. Cultivated by Ray, 1686, in his garden at Cambridge. Annual.

Spec. 4. Datura fastuosa, *purple Thorn Apple.* Syn. Stramonium fæt. pomo spinoso rotundo, semine pallido. This is an elegant plant, rising with a polished purple stalk, four feet high. The flowers are of a beautiful purple on their outside, and a satiny white within; have large swelling tubes, which spread very broad at the top, their brims having ten angles, each ending in a long slender point. The corolla is sometimes single, at others two or three flowers stand within another: the corolla is also found double, having from four to five petals within each other of equal length. The odour of the flower is agreeable at first, but if long smelt to becomes unpleasant, and is said to be *narcotic.* It is used in the East in asthmatic complaints, and is smoked through a pipe. It is a native of Egypt and the East Indies: was cultivated by Miller in 1731. Hort. Kew. Annual.

Spec. 5. Datura metel, *hairy Thorn-Apple.* Syn. Stramonium pomo spin. not. longo fl. A native of Asia, Africa, and the Canary Islands. Cultivated by Miller, in 1759. This also is smoked like the preceding species.

Spec. 6. Datura arborea, *Tree Thorn-Apple.* Syn. Stramonioides. This rises with a woody stalk to twelve or fourteen feet; produces numerous white and beautiful flowers, some of which are striped on the outside with pale yellow. Native of South America; and characterized by *Pericarps thorny, erect, ovate, leaves ovate, smooth.* The *Stem* is seldom more than two feet high, but in some soils rises to six feet. It divides into several strong irregular branches; they are hollow and covered with a fine down. *Leaves* from the forking of the stem and branches, single, scarcely six inches long, petioled, pointed, deep green on the upper surface, paler beneath and on the edges, with strong ribs or nerves, unequally sinuated and toothed about the edge, extending farther down the petiole on one side than on the other. *Petioles* round, downy, shorter than the leaves, above faintly channelled. *Flowers* single from the axils, on short peduncles, upright. *Calyx* pale green; is composed of one leaf, tubular, pentangular, and divided at the brim into five teeth. *Corolla* white, monopetalous, funnel-shaped, plicated, cut at the margin into five teeth, and furnished with a long cylindrical greenish tube: the five filaments are tapering, about the length of the calyx, adhering to the tube, and supplied with oblong flat antheræ, of a brownish yellow colour: the germen is oblong, and placed above the insertion of the corolla: the style is filiform, equal in length to the filaments, and terminated by a thick blunt stigma: the capsule is large, oval, fleshy, beset with spines, divided into cells, and four valves, which contain numerous kidney-shaped seeds of a blackish hue. At night the leaves, particularly the upper ones, rise up and inclose the flowers, which appear from July to September.

---

and one of the greatest ornaments to the gardens in Chili, where the inhabitants propagate it with great care. When the flowers are fully blown, they make a fine appearance, and a single tree will perfume the air of a large garden. Introduced by Mr. Thouin in 1783, but had been before cultivated in the Chelsea garden, and by Lord Petre, from seeds sent over by Dr. Houston. Perennial.

Spec. 7. DATURA LÆVIS, *smooth capsuled Thorn-Apple.* Syn. Datura inermis. Leaves resemble those of the D. Stramonium. Native of Africa. Introduced in 1780, by M. Thouin. Annual.

Spec. 8. DATURA INNOXIA. Syn. Stramonium fol. hyoscyami, fl. toto candido, fr. propendente rotundo, spinis innoxiis ornato. Indigenous in la Vera Cruz. Annual.*

The 2d, 4th, and 5th species may be raised by sowing the seed on a gentle hot-bed in the spring, transplanting them into the fresh ground the latter end of May. The 6th and 7th require to be kept in a stove. The 8th, in a favourable season, will rise in the spring from scattered seeds; and, if the summer prove warm, will flower, and even perfect its seed.

* Rumpf. mentions a species with flower double, fruit round, pendulous: it occasions profound sleep, and is given with advantage in dysentery.

Gerarde

Gerarde (1597) informs us that "this plant is rare and strange in England; it was brought in seed from Constantinople by the Right Hon. Lord Edward Zouch." Miller says it was probably introduced from Italy or Spain; Martyn thinks we have strong proofs of its being a native of America; for in the earth brought with plants from various parts of that country, the thorn-apple constantly springs up. In confirmation of this opinion Kalm states in his travels into North-America, "The *Datura Stramonium* grows in great quantities in all the villages; its height is different according to the soil it is in: for, in a rich soil, it grows eight or ten feet high; but, in hard and poor ground, it will seldom come up to six inches. This *Datura*, together with the *Phytolacca*, or American Nightshade, grow here in those places near the gardens, houses, and roads, which in Sweden are covered with nettles and goosefoot, which European plants are very scarce in America; but the *Datura* and *Phytolacca* are the worst weeds here, nobody knowing any particular use of them."

It has now become very common in this country, few gardens or dunghills being without it; Mr. Ray and Mr. Hudson have even placed it amongst British plants, at the same time they regard it as a doubtful native. It flowers in July. It affects the tongue with a sense of bitterness, and the smell is somewhat similar to that of the poppy. Formerly, indeed, it was supposed that by merely smelling the plant, a person would be intoxicated, "*Si tantum olfeceris Stramonium, ebrietatem fecit.*" Storck, however, who has devoted much attention to this subject, has refuted the assertion. He gathered the plant in the fields before breakfast, *summo mane jejuno ventriculo;* he rubbed the leaves and stem between his fingers, and frequently smelled them without experiencing their inebriating quality: his words are, "*Fricui folia caulemque digitis fortiter, et frequenter olfeci, percepi quidem gravem et ingratum nauseosumque odorem, verum nil temulenti, nil inebriantis adverti.*" Neither did he feel any ill effects whilst expressing the juice from the fresh leaves, or in sleeping with closed windows in the same chamber, after the operation was finished: though on waking in the morning he was affected with an obtuse pain in the head; but was in every other respect calm, and fit for business, and on breakfasting the pain left him. He evaporated eight pounds of the juice to the consistence of an extract, and neither he nor his assistant felt any inconvenience. He swallowed a small portion of the extract, and remained unaffected by it, although all the authors whom he consulted, both modern and ancient, affirmed that it produced delirium, insanity, convulsions; vulsions : " *Stramonium turbare mentem, adferre insaniam, delere ideas et memoriam, producere convulsiones.*"

Bergius states that the smell of the recent herb is powerfully narcotic, and that whilst describing the plant and chewing the leaves, he was affected with that kind of intoxication which follows smoking tobacco.* Haller ranks it amongst the worst of the narcotic plants.†

There can be no question respecting the narcotic properties of this herb, when taken in sufficient quantity ; they appear to have been known to the Chinese and the Indians, at an early period. Soon after the institution of the Royal Society, a series of inquiries was drawn up for the learned men of the East to solve. Amongst some that were curious and some that were ridiculous, it was asked, 'Whether the Indians can so prepare that stupifying herb Datura, that they make it lie several days, months, years, according as they will have it, in a man's body, without doing him any hurt, and at the end kill him, without missing half an hour's time ?" ‡ The reply returned by Sir Philberto Vernatti, resident in Batavia, was, that the " China men in this place have formerly used Datura as a fermentation, to a sort of drink much beloved by the soldiers and mariners, called *suykerbier*, which makes them raging mad, § so that it is forbidden strictly under the penalty of a great pain, to make use of the same." It was again asked, 'Whether those that be stupified by the juice of this herb Datura, are recovered by moistening the soles of their feet in fair water ?' The answer was, " No ; for I have seen divers soldiers and mariners fall into the rivers and ditches, being stupified by their drink aforesaid, who were rather worse when they were taken out than better."

Haller states that it causes delirium, mania, convulsions, paralysis, cold sweats, vehement thirst, tremors. A man

* " Odor recentis herbæ narcoticus, adeo fortis et afficiens, ut, cum plantam describerem, ex odore narcotico, et ex eo quod masticaveram folii, frustum, temulentus quasi evaserim, haud secus ac si intuetus fumum tabaci hausissem. Materia Med. tom. i. 121.

† " Ex deterioribus plantis narcoticus est. Somnum facit adeo profundum, ut impune pudicitia puellæ violari possit, quæ hoc toxicum sumserit." (Hist. Stirp. Indigen. Helvet.)

‡ Spratt's Hist. Royal Soc.

§ The delirium produced by Stramonium was very agreeable to the Indians. Kæmfer, as quoted by Paller, observes, " Deliria facit apud Indos grata equidem, reformata hactenus planta virosæ efficacia. Nam omnino Stramonium est Indorum Datura, confirmantibus Missionariis Danicis."

who who took a decoction of the fruit was sad, speechless; he lost the use of his limbs, and at length became furiously mad. These effects, however, depend upon the dose. A small quantity occasions a pleasant kind of delirium which disposes a person to be very good natured, and willingly part with his property. Cunning knaves have availed themselves of this quality with great success. Garzia D'all'Horto in his history of Oriental aromatic simples used in medicine, mentions that the Datura grows in Malabar, and that when rogues design to rob a person, they mix some of the flowers with his food; those who partake of it lose their senses, are seized with violent laughter, and readily suffer themselves to be pillaged. This state continues about twenty-four hours. The means recommended by this author for recovering such unfortunate victims, are emetics, purgatives, clysters, friction, and bleeding from the foot.*

The following extract from Sauvages describes some very similar effects from the seeds of the Stramonium infused in wine, and given to travellers by a gang of robbers; the chief of whom confessed, that before he had ascertained the proper dose, several persons died from the violent effects of the poison. "Nuper Monspelii plures nefarii capti sunt, qui viatores expoliaverant propinato vino, in quo semina daturæ Stramonii contrita et infusa fuerant; fassus est autem eorum dux plures ab eo veneno interemptos esse, cum veram veneni dosim ignorabat, atque ea dosis nimia lethargum inducebat; dosis vero minor tantummodo paraphrosynem minime lethalem in bene multis, quorum aliquot ipse interrogavi, creaverat." Nosolog. Method. Pars. II. p. 330. Various stories of this sort are related by different writers, both ancient and modern. A provincial paper, even very recently, detailed a

---

* "Quandro i ladri voglion rubbare alcuno, mettono di quei fiori ne i cibi, e glie li danno a mangiare; percioche tutti coloro che ne mangiano perdono il cervello, e vengono in grandissime risa, et in gran liberalita; concendendo di propria volonta, che ogni uno loro rubbi. Suole tale alienatione di mente durare per spatio de ventiquattro hore. La prima cosa, che si de far per curarli, si de provocare il vomito, perche buttino quanto hanno nello stomaco insieme col cibo; dosso si deono evacuare, e far con cristeri gagliarde diversione, e cosi ancora con forti, e gagliarde fregaggioni alle gambe poco piu sopra del piede, e tal' hora anco trar loro sangue dalla vena del piede. Con questa sorte di remedii giamai alcuno di miei amalati si mori; ma tutti gratia al Signore, sono in termine di ventiquattro hore quariti." Dell' Historia dei Simplici Aromat, &c. &c. (Venetia, MDLXXXIX.)

successful instance of this kind of roguery, and the practice was common in France a few years since. It is not very improbable that ale-brewers are acquainted with the pleasant properties of this herb, as a small dose occasions an agreeable degree of intoxication, which soon goes off without occasioning any bad consequences.

The Turks at an early period appreciated the effects of Stramonium on the brain. According to Velschius, the terms *Dotter*, *Datura*, *Tatula*, are corruptions of the Turkish *Taltan*, or *Tsaltan*, *Tatalat*, or *Tsatsalat*. Lindelstolpe mentions that the Turkish women who were unfaithful were accustomed to give it their husbands, who, in consequence, were stupified, and their intellects became disordered, whilst their wives enjoyed the embraces of their favourites without restraint. Faber, in his very curious and learned work on Strychnomania, even makes the luckless husbands witness their shame, and laugh at the joke.*

*(To be continued.)*

---

* As this treatise is very rare, an extract from the author's account of Datura will probably interest many readers.

" Celebre autem est maleficium apud Indos, quando nimirum sive fœminæ maritis impositurae zelotypis, decoctum Daturæ illis propinant, quo fit, ut apertis oculis consortium nequitiam et turpitudinem propriam inspicientes, mente tamen turbati, cachinno tantum seipsos deludant, ac subsannatium morsus probent, cum

Certant ingenio quisnam solertius instet
Aut vota lusum vertat in contrarium.
Dum modo commonstrant ficum prædulcibus uvis
Compressa qualem pollice efformat manus
Aut miseri frontem pulsant resonante talitro
Unaque tractos capitis evellunt pilos.

Quo etiam modo servi nequam heris pocula solent infuere, ac palantium expilare thesauros, cujusmodi historiæ ludicræ pænes authores leguntur citatos.

Hujusmodi sine dubio fuerunt potiones illæ, quibus tres nebulones Augustæ Vindel. utebantur, ad inducendum hominibus vesaniam somnumque, securius post modum omnem substantiam eorum asportantes, quibus anno 1429, condignum præmium, furca, contigit."

